# Docetaxel increases the risk of severe infections in the treatment of non-small cell lung cancer: a meta-analysis

**DOI:** 10.18632/oncoscience.444

**Published:** 2018-08-22

**Authors:** Qingcheng Du, Guanming Jiang, Silu Li, Yong Liu, Zunnan Huang

**Affiliations:** ^1^ School of Pharmacy, Guangdong Medical University, Dongguan, Guangdong 523808, China; ^2^ Department of Medical Oncology, Dongguan People’s Hospital, Dongguan, Guangdong 523018, China; ^3^ School of Basic Medicine, Guangdong Medical University, Dongguan, Guangdong 523808, China; ^4^ Key Laboratory for Research and Development of Natural Drugs of Guangdong Province, Zhanjiang, Guangdong 524023, China; ^5^ Key Laboratory for Medical Molecular Diagnostics of Guangdong Province, Dongguan Scientific Research Center, Guangdong Medical University, Guangdong 523808, China

**Keywords:** docetaxel, infections, non-small cell lung cancer, mechanism, systematic review

## Abstract

The purpose of this study was to determine whether docetaxel increases the risk of severe infections in patients with non-small cell lung cancer. A thorough literature search of the PubMed, EMBASE and Cochrane Central Register of Controlled Trials databases was performed (up to February 28, 2017) without any language restrictions. In addition, we searched the www.clinicaltrials.gov website and checked each reference listed in the included studies, relevant reviews and guidelines. We also included randomized controlled trials that reported severe infections in patients with non-small cell lung cancer who were administered docetaxel. A meta- analysis was conducted using relative risk and random effects models in Stata 14.0 software. Sensitivity analysis and meta-regression were performed using Stata 14.0 software. We identified 354 records from the initial search, and this systematic review ultimately included 43 trials with 12,447 participants. The results of our meta- analysis showed that docetaxel increased the risk of severe infections [relative risk: 2.10, 95% confidence interval: 1.51-2.93, *I*^2^ = 69.6%, *P* = 0.000]. Meta-regression analysis indicated that the type of intervention was a major source of heterogeneity. Our systematic review and meta-analysis suggest that docetaxel is associated with the risk of severe infections.

## INTRODUCTION

Lung cancer is a respiratory disease, one of the most common types of cancer, and the leading cause of cancer-associated death worldwide. Each year, there are more than 1.3 million new cases of lung cancer [1]. According to pathological classification systems, lung cancer is divided into small cell lung cancer and non-small cell lung cancer (NSCLC) [2, 3]. NSCLC accounts for approximately 85% of lung cancer cases [4] and consists of three different subtypes: squamous cell carcinoma, large cell carcinoma and adenocarcinoma [2]. Most patients with NSCLC present with advanced or metastatic disease, and the main metastatic sites of NSCLC are the brain, bone, liver and adrenal glands [4, 5]. The best treatment option for patients with NSCLC is surgery, and approximately 20% of these patients are good candidates for potentially curative resection [6]. However, even for patients who undergo a complete resection, the 5-year survival rate is only 40% [7]. Thus, NSCLC is a serious threat to human health.

The current common chemotherapy agents include cisplatin, carboplatin, gemcitabine, pemetrexed, paclitaxel and docetaxel [8]. Docetaxel, a paclitaxel derivative, is a second-generation taxane anticancer agent. Its main mechanisms of action include promoting tubulin binding, inhibiting its agglomeration, and significantly stabilizing its conformation, leading to cell cycle arrest during G2/M phase and consequent anti-tumor effects [9]. Compared to paclitaxel, docetaxel has better water solubility and fewer toxic side effects [10]. Moreover, docetaxel can reduce Bcl2 (anti-apoptosis) gene expression and promote the expression of the cell cycle regulator p27, which results in cancer cell apoptosis [11]. Docetaxel has synergistic effects with other chemotherapy agents in prostate cancer [12] and has been approved as a first-line treatment combined with cisplatin, as a single-dose second-line treatment, or as a single-dose maintenance treatment for advanced NSCLC patients in many countries [13–15]. Single- dose docetaxel treatment is promising as first-line therapy for NSCLC patients with a poor performance status [15]. Furthermore, docetaxel is effective as a second-line therapy for metastatic NSCLC, extending progression-free survival and overall survival [16, 17]. Due to its beneficial effects and good tolerability, docetaxel plays an important role in the treatment of NSCLC [18]. However, some studies [19–22] have shown that docetaxel can increase the risk of adverse reactions, such as neutropenia, febrile neutropenia and anemia, in patients with advanced NSCLC. These adverse reactions have attracted attention from the research community.

Previous publications have explored the link between infections and docetaxel treatment [19, 21]. For instance, some randomized controlled trials [23–25] reported that docetaxel increased the risk of severe infections in patients with NSCLC, while others [26–28] found that severe infections were not associated with docetaxel. Thus, a definitive conclusion has not been reached. Additionally, no previous systematic review or meta-analysis has been conducted to comprehensively assess the risk of severe infections (≥grade 3) in docetaxel-treated patients with NSCLC. Therefore, we performed this study to determine whether docetaxel increases the risk of severe infections (≥grade 3) in patients with NSCLC.

## RESULTS

### Search results and trial descriptions

**Figure 1:**
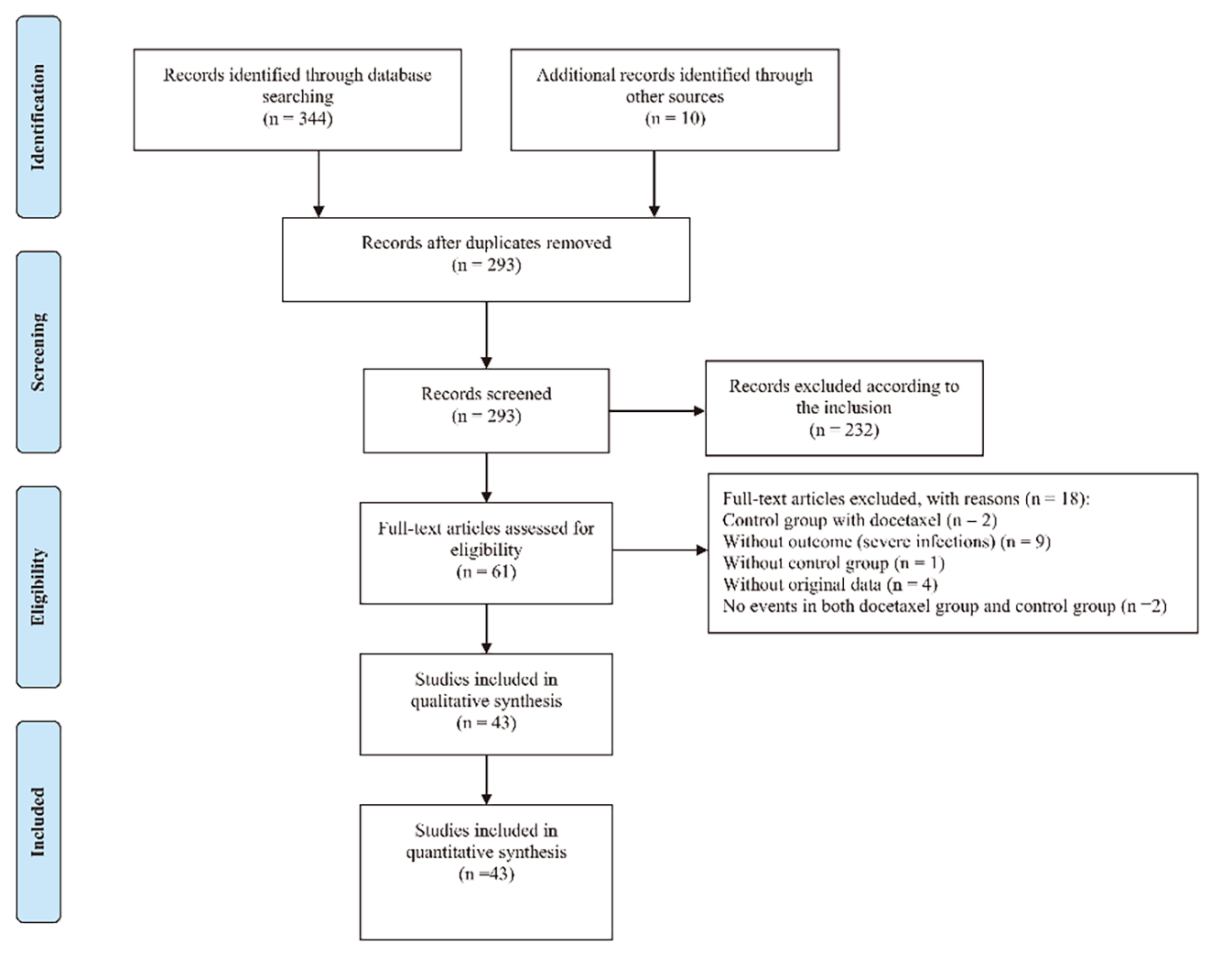
Flow diagram of study selection of all included randomized controlled trials

The initial search identified 354 records and bibliographies of relevant reviews from electronic databases, including studies and guidelines, generated prior to February 28, 2017. A total of 293 trials were obtained after duplicates were removed. According to the inclusion and exclusion criteria, 232 trials were eliminated after title and abstract review. Thus, a total of 61 trials remained, and the full-text articles corresponding to these trials were obtained to further assess eligibility. After a careful review of these articles, 18 were excluded due to ineligibility or incomplete data. In the end, this systematic review included 43 studies with 12,447 participants, including 40 journal articles [23–62] and three trials (NCT00520676, NCT00191139, and NCT01204697) from the clinical trial registration website (https://clinicaltrials.gov). Details of the study selection are shown in (Figure 1).

The main characteristics of all 43 trials included in this meta-analysis are listed in (Table 1). Among all the included trials, 19 were phase II trials, and 24 were phase III trials. The sample size of these trials varied from 51 to 1,446, and all participants were older than 50 years of age. While a 75 mg/m^2^ dose was typically used in the docetaxel-only group, the control group was given other drugs or supportive care. Most studies focused on stage III- IV disease and on tumor histologies of adenocarcinoma, squamous cell carcinoma, bronchioalveolar carcinoma, and large cell carcinoma.

**Table 1:** Characteristics of each included study

Study and year	Phase	Race	Intervention	Disease stage	Tumor histology	Median follow-up
Aerts JG 2013	II	NR	D: erlotinib 150 mg + docetaxel 75 mg/m^2^ + pemetrexed 500 mg/m^2^ N: erlotinib 150 mg	Stage IB, IIIB, IV	Squamous cell carcinoma, adenocarcinoma, large cell, undifferentiated, bronchoalveolar, plavelsel, other, unknown	19 months
Barlesi F 2015	III	NR	D: cisplatin 75 mg/m^2^ + docetaxel 75 mg/m^2^ N: cisplatin 75 mg/m^2^ + gemcitabine 1250 mg/m^2^	Stage IB–III	Adenocarcinoma and squamous cell carcinoma	20.2 months
Booton R 2006	III	NR	D: docetaxel 75 mg/m^2^, carboplatin (AUC = 6) N: mitomycin 6 mg/m^2^, ifosfamide 3 g/m^2^ + cisplatin 50 mg/m^2^ or mitomycin 6 mg/m^2^, vinblastine 6 mg/m^2^ + cisplatin 50 mg/m^2^	Stage III, IV	Squamous cell carcinoma, adenocarcinoma, large cell, undifferentiated non-small lung cancer	17.4 months
Borghaei H 2015	III	White, Black, Asian, Other	D: docetaxel 75 mg/m^2^ N: nivolumab 3 mg/kg	Stage IIIB, IV	Squamous cell carcinoma	9.2 months
Ciuleanu T 2012	III	Caucasian, Asian, other	D: chemotherapy (docetaxel 75 mg/m^2^, pemetrexed regimens) N: erlotinib 150 mg	Stage IIIB, IV	Adenocarcinoma, squamous cell carcinoma, other	27.9 months
Cufer T 2006	II	Caucasian, Black, Asian, Oriental, Other	D: docetaxel 75 mg/m^2^ N: gefitinib 250 mg	Stage IIIB, IV	NR	9.4 months
Douillard JY 2005	II	NR	D: docetaxel 75 mg/m^2^ + cisplatin 100 mg/m^2^ N: vinorelbine 30 mg/m^2^ + cisplatin 100 mg/m^2^	Stage IV	Squamous, adenocarcinoma, large cell carcinoma, undifferentiated lung cancer, mixed histological subtype	8.8 months
Edelman MJ 2004	II	NR	D: docetaxel 75–100 mg/m^2^ + cisplatin 100 mg/m^2^ + vinorelbine 25 mg/m^2^ N: carboplatin 5.5 mg/ml/min + gemcitabine 1,000 mg/m^2^ + paclitaxel 225 mg/m^2^	Stage IIIB, IV	Squamous, adenocarcinoma, large cell carcinoma, other	NR
Esteban E 2008	II	NR	D: gemcitabine 1,000 mg/m^2^ + docetaxel 35 mg/m^2^ N: gemcitabine 1,000 mg/m^2^ + vinorelbine 25 mg/m^2^	Stage IIIB, IV	Squamous, adenocarcinoma, large cell carcinoma	9.5 months
Fanucchi MP 2006	II	White, Black, Asian/Pacific Islander, Hispanic, Other	D: bortezomib 1.3 mg/m^2^ + docetaxel 75 mg/m^2^ N: bortezomib 1.5 mg/m^2^	Stage IIIB, IV	Adenocarcinoma, squamous cell, bronchioalveolar, large cell, adenosquamous, epithelioid carcinoma, unknown	NR
Fehrenbacher L 2016	II	NR	D: docetaxel 75 mg/m^2^ N: atezolizumab 1200 mg	Stage IIIB, IV, recurrent	Non-squamous and squamous	14.8 months
Fossella F 2003	III	NR	D: docetaxel 75 mg/m^2^ + cisplatin 75 mg/m^2^ + carboplatin 6 mg/mL/min N: vinorelbine 25 mg/m^2^ + cisplatin 100 mg/m^2^	Locally advanced, stage IIIB, metastatic, stage IV	Adenocarcinoma, large cell, squamous cell, bronchioalveolar carcinoma, other	10 months
Garassino MC 2013	III	White, Asian	D: docetaxel 75 mg/m^2^ or 35 mg/m^2^ N: erlotinib 150 mg	Locally advanced or metastatic	Adenocarcinoma, large cell, squamous cell, bronchioalveolar carcinoma, other	33 months
Gebbia V 2010	II	NR	D: cisplatin 75 mg/m^2^ + docetaxel 75 mg/m^2^ N: cisplatin 80 mg/m^2^ + vinorelbine 30 mg/m^2^	Stage IIIB, IV	Squamous, adenocarcinoma, large cell carcinoma	NR
Georgoulias V 2005	III	NR	D: gemcitabine 1,000 mg/m^2^ + docetaxel 100 mg/m^2^ N: vinorelbine 30 mg/m^2^ + cisplatin 80 mg/m^2^	Stage IIIB, IV	Squamous, adenocarcinoma, large cell carcinoma, undifferentiated, mixed	9.0 months
Gridelli C 2016	II	NR	D: erlotinib 150 mg + docetaxel 75 mg/m^2^ N: erlotinib 150 mg	Stage IIIB, IV	Squamous cell carcinoma	NR
Hanna N 2004	III	NR	D: docetaxel 75 mg/m^2^ N: pemetrexed 500 mg/m^2^	Stage III, IV	Adenocarcinoma, squamous cell carcinoma	7.5 months
Juan Ó 2015	II	NR	D: erlotinib 150 mg + docetaxel 75 mg/m^2^ N: erlotinib 150 mg	Stage IIIB, IV	Adenocarcinoma, squamous cell, large cell carcinoma, non-small cell carcinoma	6.2 months
Karampeazis A 2011	III	NR	D: docetaxel 38 mg/m^2^ N: vinorelbine 25 mg/m^2^	Stage IIIB, IV	Squamous, adenocarcinoma, large cell carcinoma. undifferentiated	40.5 months
Karayama M 2013	II	NR	D: docetaxel 60 mg/m^2^ N: pemetrexed 500 mg/m^2^	Stage IIIB, IV	adenocarcinoma, other	16.8 months
Kawaguchi T 2014	III	NR	D: docetaxel 60 mg/m^2^ N: erlotinib 150 mg	Stage IIIB, IV	Adenocarcinoma, squamous cell carcinoma, other	8.9 months
Kawahara M 2013	II	NR	D: docetaxel 60 mg/m^2^ + carboplatin (AUC = 6) N: paclitaxel 200 mg/m^2^ + carboplatin (AUC = 6)	Stage IIIB, IV	Squamous cell, adenocarcinoma, large cell carcinoma	15.8 months
Kim ES 2008	III	White, Asian, Black, Other	D: docetaxel 75 mg/m^2^ N: gefitinib 250 mg	Stage 0/I, IIa/IIb, IIIa, IIIb, IV, not recorded	Adenocarcinoma, bronchoalveolar, squamous cell, large cell, mixed, undifferentiated, other	7.6 months
Krzakowski M 2010	III	NR	D: docetaxel 75 mg/m^2^ N: vinflunine 320 mg/m^2^	Stage IIIB, IV, other	Squamous cell, adenocarcinoma carcinoma, other	NR
Kubota K 2015	III	Japanese	D: docetaxel 60 mg/m^2^ + cisplatin 80 mg/m^2^ N: S-1 80 mg/m^2^/day + cisplatin 60 mg/m^2^	Stage IIIB, IV, postoperative, recurrence	Adenocarcinoma, squamous cell, large cell carcinoma, adenosquamous, other	NR
Lilenbaum R 2006	II	White, Black, Asian/Pacific Islander, Hispanic, Other	D: irinotecan 60 mg/m^2^ + docetaxel 35 mg/m^2^ + celecoxib 400 mg N: irinotecan 100 mg/m^2^ + gemcitabine 1,000 mg/m^2^ + celecoxib 400 mg	Stage IIIB, IV	NR	NR
Mattson KV 2003	III	NR	D: docetaxel 100 mg/m^2^ N: no chemotherapy	Stage IIIA T3, IIIA, N2, IIIB	Squamous cell, adenocarcinoma, large cell carcinoma, other	NR
Movsas B 2010	II	Caucasian, Asian, Other	D: gemcitabine 1,000 mg/m^2^ + docetaxel 75 mg/m^2^ N: gemcitabine 1,000 mg/m^2^	Stage IIIA, IIIB, unavailable	Squamous, adenocarcinoma, large cell, mixed, other	41.5 months
NCT00191139	II	Caucasian, Black Asian, Hispanic	D: docetaxel 75 mg/m^2^ + gemcitabine 1,000 mg/m^2^ N: gemcitabine 1,000 mg/m^2^	Stage III	NR	NR
NCT00520676	III	Taiwanese, Mexican, Brazilian, Australian, Chinese, Korean	D: docetaxel 75 mg/m^2^ + carboplatin (AUC = 5) N: pemetrexed 500 mg/m^2^ + carboplatin (AUC = 5)	Stage IIIB, IV	Adenocarcinoma, large cell carcinoma	NR
NCT01204697	II	NR	D: docetaxel 75 mg/m^2^ plus erlotinib 150 mg N: erlotinib 150 mg	locally advanced (stage IIIB), metastatic (stage IV), recurrent	Squamous cell carcinoma	NR
Nishino K 2015	II	NR	D: docetaxel 60 mg/m^2^ + bevacizumab 15 mg/kg N: S-1 40 mg/m^2^ + bevacizumab 15 mg/kg	Stage IIIB, IV	Adenocarcinoma, unclassified non-small cell carcinoma	14.6 months
Park CK 2017	III	NR	D: docetaxel 60 mg/m^2^ + cisplatin 70 mg/m^2^ N: pemetrexed 500 mg/m^2^ + cisplatin 70 mg/m^2^	Stage IIIB, IV	Adenocarcinoma, large cell carcinoma	NR
Pérol M 2002	II	NR	D: docetaxel 100 mg/m^2^ + cisplatin 100 mg/m^2^ + vinorelbine 30 mg/m^2^ N: cisplatin 80 mg/m^2^ + vinorelbine 30 mg/m^2^	Stage IV	Epidermoid, adenocarcinoma, large cell carcinoma	103 weeks
Pujol JL 2005	III	NR	D: gemcitabine 1,000 mg/m^2^ + docetaxel 85 mg/m^2^ N: cisplatin 100 mg/m^2^ + vinorelbine 30 mg/m^2^	Stage IIIB, IV	Squamous cell, adenocarcinoma, large cell carcinoma	NR
Ramlau R 2006	III	White, Oriental, Black, Other	D: docetaxel 75 mg/m^2^ N: topotecan 2.3 mg/m^2^	Stage IIIB, IV	Adenocarcinoma, squamous cell large cell carcinoma, other	NR
Rittmeyer A 2017	III	White, Asian, Black, Other	D: docetaxel 75 mg/m^2^ N: atezolizumab 1,200 mg	Stage IIIB, IV	Non-squamous, squamous carcinoma	21 months
Rocha Lima CMS 2004	II	NR	D: gemcitabine 1,000 mg/m^2^ + docetaxel 40 mg/m^2^ N: gemcitabine 1,000 mg/m^2^ + irinotecan 100 mg/m^2^	Stage IIIB, IV	Adenocarcinoma, squamous cell, undifferentiated large cell, bronchoalveolar, undifferentiated non-small cell carcinoma	20 months
Rodrigues-Pereira J 2011	III	East Asian, Caucasian, Hispanic, African	D: carboplatin (AUC = 5) + docetaxel 75 mg/m^2^ N: carboplatin (AUC = 5) + pemetrexed 500 mg/m^2^	Stage IIIB, IV	Adenocarcinoma, large cell carcinoma	23.9 months
Rosell R 2012	III	NR	D: cisplatin 75 mg/m^2^ + docetaxel 75 mg/m^2^ + gemcitabine 1250 mg/m^2^ N: erlotinib 150 mg	Stage N3, IIIA, IIIB, IV	Adenocarcinoma, bronchoalveolar, squamous cell carcinoma, large cell, other	18.9 months
Roszkowski K 2000	III	NR	D: docetaxel 100 mg/m^2^ + best supportive care N: best supportive care	Stage IIIB, IV	Squamous cell, adenocarcinoma or undifferentiated, large cell carcinoma	NR
Sun Y 2013	III	Chinese	D: docetaxel 75 mg/m^2^ N: pemetrexed 500 mg/m^2^	Stage IIIB, IIIA, IV	Adenocarcinoma, squamous cell, mixed cell carcinoma	NR
Wu YL 2013	III	Chinese	D: docetaxel 75 mg/m^2^ N: pemetrexed 500 mg/m^2^	Stage III–IV	Adenocarcinoma, other (mixed cell carcinoma)	NR

Notes: AUC: area under the curve; NR: not reported.

**Figure 2:**
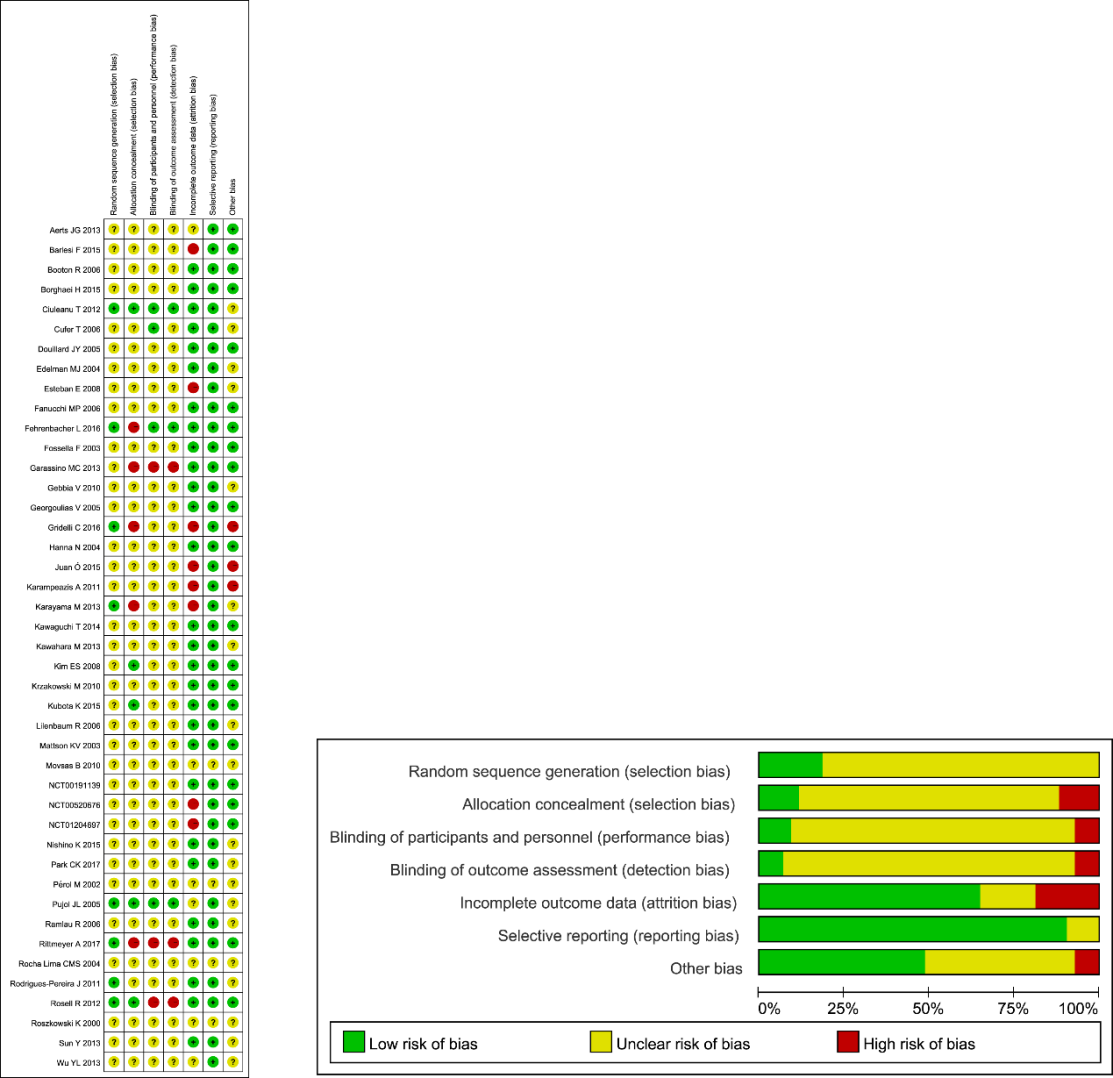
Risk of bias of each included trial as assessed using the risk of bias assessment tool

The risk of bias in all the included trials is summarized in (Figure 2). With respect to random sequence generation, allocation concealment, blinding of participants and personnel, and blinding of outcome assessment, most trials showed an unclear risk of bias according to the authors’ judgment. On the other hand, with respect to incomplete outcome data, selective reporting and other biases, most trials showed a low risk of bias.

### Relative risk (RR) of severe infections

**Figure 3:**
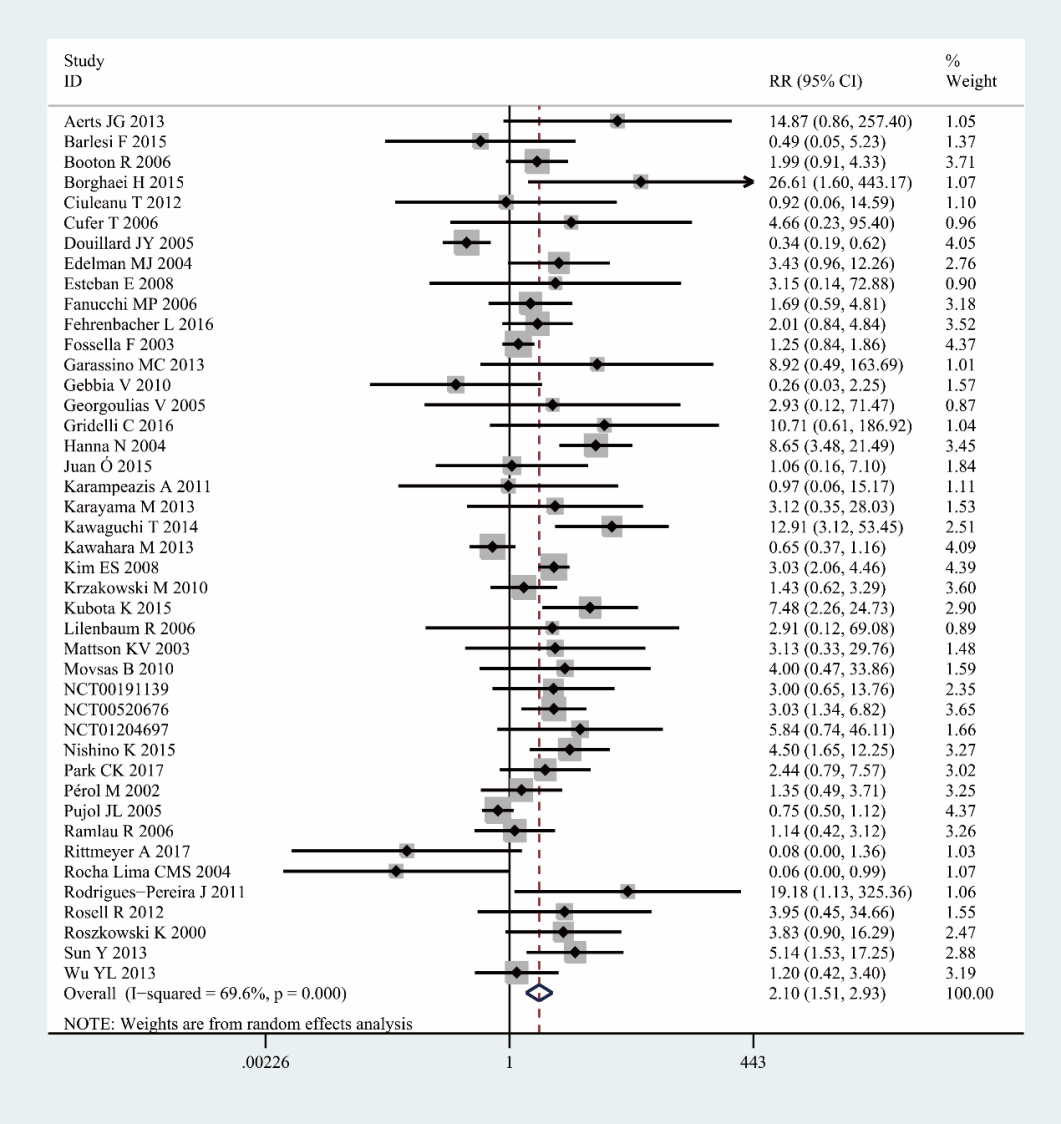
Relative risk and 95% confidence interval for severe infections between the docetaxel group and the control group

The meta-analysis of 43 randomized controlled trials indicated that docetaxel was related to a significant increase in the risk of severe infections. The RR of severe infections in NSCLC patients treated with docetaxel compared to that in controls was 2.10 (95% confidence interval (CI): 1.51-2.93, *I^2^* = 69.6%, *P* = 0.000) (Figure 3). The heterogeneity among studies was significant (*I^2^* > 50%), and thus, the random effects model was selected.

### Subgroup analysis by age

In the ≥60 years age subgroup from a total of 24 trials and 6,835 participants, 391 patients had severe infections (279 in the docetaxel group and 112 in the control group), for a RR of 2.03 (95% CI: 1.37-3.01, *P* = 0.000). In the younger subgroup (<60 years) from a total of 19 trials and 5,612 participants, 443 patients had severe infections (288 in the docetaxel group and 155 in the control group), for a RR of 2.12 (95% CI: 1.20-3.74, *P* = 0.010). Thus, the subgroup analysis by age showed that docetaxel consistently increased the risk for severe infections in NSCLC patients (Table 2). The forest plot of this subgroup analysis is shown in (Supplementary Figure 1).

### Subgroup analysis by intervention type

Subgroup analysis was also performed on the intervention type (docetaxel alone versus docetaxel with other interventions). The RR of severe infections was 3.26 (95% CI: 2.12-5.00, *P* = 0.000) for the docetaxel alone group from a total of 15 trials and 4,865 individuals, 318 of whom had severe infections (250 in the docetaxel group and 68 in the control group). The RR was 1.62 (95% CI: 1.09-2.39, *P* = 0.016) for the group of patients who received additional drug interventions from a total of 28 trials and 7,582 individuals, 516 of whom had severe infections (317 in the docetaxel group and 199 in the control group). The subgroup analysis by intervention type again showed that docetaxel increased the risk of severe infections in NSCLC patients (Table 2). The forest plot of this subgroup analysis is shown in (Supplementary Figure 2).

### Subgroup analysis by docetaxel dosage

In this subgroup analysis, we divided the 43 trials into two subgroups according to the docetaxel dosage (≥75 mg/m^2^ versus <75 mg/m^2^). In the higher dose subgroup (≥75 mg/m^2^) from 33 trials and 10,857 participants, 700 patients had severe infections (469 in the docetaxel group and 231 in the control group), yielding a RR of 2.00 (95% CI: 1.40-2.87, *P* = 0.000). For the lower dose subgroup (<75 mg/m^2^) from 10 trials and 1,590 participants, 134 patients had severe infections (98 in the docetaxel group and 36 in the control group), for a RR of 2.34 (95% CI: 0.91-6.03, *P* = 0.077). Thus, the subgroup analysis by docetaxel dosage showed that a higher dose of docetaxel (≥75 mg/m^2^) increased the likelihood of severe infections in NSCLC patients, while a lower dose of docetaxel (<75 mg/m^2^) did not have a harmful effect on the development of severe infections (Table 2). The forest plot of this subgroup analysis is shown in (Supplementary Figure 3).

### Subgroup analysis by trial phase

Finally, we performed a subgroup analysis according to trial phase (phase II versus III). The RR of severe infections was 1.69 (95%: 0.97-2.96, *P* = 0.064) from a total of 19 phase II trials and 2,138 individuals, including 217 with severe infections (125 in the docetaxel group and 92 in the control group). For the 24 phase III trials of 10,309 individuals, 617 patients had severe infections (442 in the docetaxel group and 175 in the control group), yielding a RR of 2.45 (95% CI: 1.63-3.69, *P* = 0.000). The subgroup analysis by trial phase illustrated that docetaxel did not contribute to the risk of severe infections in NSCLC patients during phase II trials but did increase the risk of severe infections in NSCLC patients during phase III trials (Table 2). The forest plot of this subgroup analysis is shown in (Supplementary figure 4).

**Table 2:** Subgroup analysis results

Subgroup	Trials (n)	No. of severe infections		No. of participants		RR (95% CI)	*I*^2^	*P*-value
		Docetaxel	Control	Docetaxel	Control			
Age								
≥60	24	279	112	3,642	3,193	2.03 (1.37, 3.01)	53.8%	0.000
<60	19	288	155	2,842	2,770	2.12 (1.20, 3.74)	79.7%	0.010
Intervention								
Docetaxel without other interventions	15	250	68	2,438	2,427	3.26 (2.12, 5.00)	37.3%	0.000
Docetaxel with other interventions	28	317	199	4,046	3,536	1.62 (1.09, 2.39)	67.2%	0.016
Dose of docetaxel								
≥75 mg/m^2^	33	469	231	5,679	5,178	2.00 (1.40, 2.87)	68.6%	0.000
<75 mg/m^2^	10	98	36	805	785	2.34 (0.91, 6.03)	75.3%	0.077
Phase of trial								
II	19	125	92	1,083	1,055	1.69 (0.97, 2.96)	64.7%	0.064
III	24	442	175	5,401	4,908	2.45 (1.63, 3.69)	70.6%	0.000

In addition, we performed a subgroup analysis according to disease stage (stages IIB and IV only vs mixed stages). The forest plot of this subgroup analysis is shown in (Supplementary figure 5).

### Meta-regression analysis

The heterogeneity among the included studies was significant. Thus, we performed a meta-regression analysis to explore the source of heterogeneity. The meta- regression analysis was performed using Stata 14.0 to analyze age, intervention type, docetaxel dosage, and trial phase. The result of our meta-regression analysis showed that age, docetaxel dosage and trial phase were not sources of heterogeneity (*P* = 0.983, *P* = 0.960, and *P* = 0.238, respectively), while heterogeneity was indeed introduced by intervention type (*P* = 0.028).

### Sensitivity analysis

**Figure 4:**
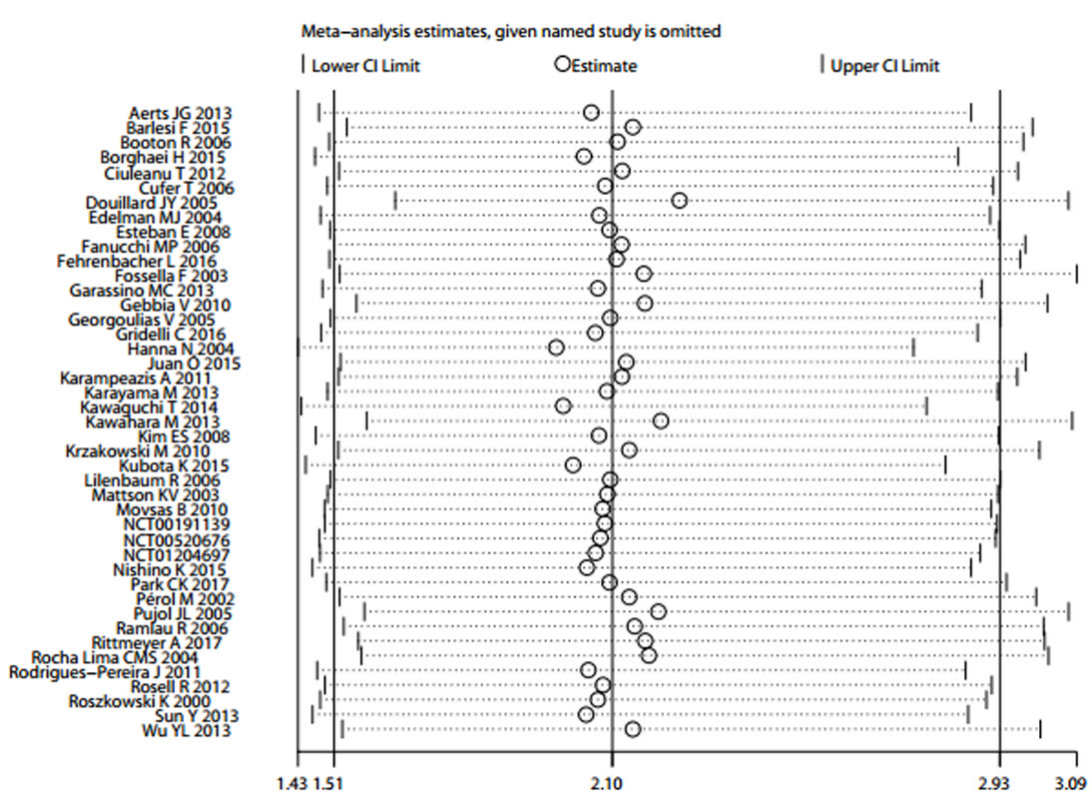
Sensitivity analysis of severe infections in patients with non-small cell lung cancer treated with docetaxel

The sensitivity analysis showed that the pooled results were not significantly changed after deleting each trial, which confirmed the rationality and reliability of our meta-analysis (Figure 4).

### Publication bias

According to the result of Begg’s test (*P* = 0.900), no publication bias existed in this meta-analysis.

## DISCUSSION

To the best of our knowledge, this study is the first systematic review and meta-analysis to assess the risk of severe infections (≥grade 3) in docetaxel-treated patients with NSCLC. A few meta-analyses have compared docetaxel to other drugs in the treatment of NSCLC. For example, Di BS et al. showed that docetaxel had a similar efficacy to pemetrexed in patients with advanced NSCLC, while docetaxel resulted in a higher rate of febrile neutropenia (grade 3-4), neutropenia, diarrhea, leukocytes, and alopecia toxicity and a lower rate of thrombocytopenia (grade 3-4) than did pemetrexed [21]. He X et al. also reported that docetaxel could improve both progression- free survival and the overall response rate as a first- line treatment for patients with advanced NSCLC compared to vinca alkaloid; furthermore, docetaxel had a lower rate than vinca alkaloid of hematological and non-hematological toxicity (grade 3/4) [22]. Although these previous meta-analyses assessed the efficacy and some adverse events of docetaxel in the treatment of NSCLC, they did not comprehensively assess the risk of severe infections (≥grade 3) in docetaxel-treated patients, thereby providing the rationale for conducting this systematic review.

Our systematic review and meta-analysis, which included 43 randomized controlled trials consisting of 12,447 participants, showed that docetaxel significantly increased the risk of severe infections (≥grade 3) in patients with NSCLC. In addition to the overall analysis, we performed subgroup analyses on such factors as age, type of intervention, docetaxel dosage, and trial phase. While subgroup analyses based on age and intervention type gave results consistent with those of the overall meta-analysis, the subgroup analyses based on docetaxel dosage and trial phase produced slightly different outcomes. On the one hand, the docetaxel dosage ≥75 mg/m^2^ and phase III trial subgroups showed that docetaxel increased the risk of severe infections in NSCLC patients; on the other hand, no significant differences in severe infections were observed between the docetaxel group and the control group for the <75 mg/m^2^ dosage and phase II trial subgroups. Although these latter subgroups showed some differences from the overall results, they were interpretable. It was previously reported that febrile neutropenia and non-neutropenic infections were associated with docetaxel dosage [63]. While a high dose of medicine will always carry a high risk of side effects, a lower dose (<75 mg/m^2^) is more likely to have a neutral impact on the risk of severe infections. Additionally, sample size may have influenced the results of the subgroup analysis, leading to the differences in the pooled results between the subgroups including 19 phase II trials and 2,138 participants and 24 phase III trials and 10,309 participants. Additionally, to assess the potential effect of drugs used in combination with docetaxel, a subgroup analysis was performed to differentiate studies testing docetaxel alone versus studies testing docetaxel-based combinations. Both the docetaxel alone subgroup and the docetaxel with other interventions subgroup revealed that docetaxel is associated with an increased risk of severe infections.

Although the results from this meta-analysis clearly indicate that docetaxel treatment is significantly associated with the risk of severe infections, the mechanism underlying these docetaxel-induced adverse reactions remains unknown. Kotsakis A et al. showed that most patients undergoing docetaxel treatment with nonneutropenic infections had an extremely low absolute number of peripheral blood lymphocytes, indicating that these patients likely developed impaired immune function [64]. Therefore, docetaxel-related severe infections are possibly due to a cytotoxic effect of docetaxel on the immune system. However, further studies are required to thoroughly explore this possible mechanism of docetaxel- associated infection.

As a result of recent studies [31, 37], immunotherapy has become a promising therapy for NSCLC and has provided an important breakthrough in the treatment of lung cancer. Common immunotherapy agents include anti-programmed cell death protein-1 (anti-PD-1), anti-programmed cell death-ligand 1 (anti-PD-L1) and cytotoxic T-lymphocyte antigen-4 (CTLA-4) therapeutics. For example, two anti-PD-1 therapies (nivolumab and pembrolizumab) and one anti-PD-L1 agent (atezolizumab) are in the advanced stages of development as treatments for advanced or metastatic NSCLC [65]. In clinical trials, nivolumab was shown to significantly improve overall survival, response rate, and progression-free survival and to have a lower incidence of treatment-related adverse events (grade 3 or 4) than docetaxel in cases of advanced squamous cell NSCLC [31]. Atezolizumab also led to much better survival outcomes and a lower incidence of treatment-related adverse events (grade 3–4) than docetaxel in the treatment of NSCLC patients [37]. Therapeutic strategies for lung cancer will likely change in response to the successful clinical application of PD-1 immune checkpoint blockade [65]. Although immunotherapy has become a promising therapy for NSCLC, its development still faces several significant challenges. First, immunotherapy cannot be used as first-line treatment and does not work well with other effective treatments. Second, immunotherapy with anti-PD-1/anti-PD-L1 agents may increase the risk of hyper-progressive disease [66, 67], and PD-1 inhibitors might increase the risk of pneumonitis compared to common chemotherapy agents [68]. Third, the high cost of immunotherapies such as anti- PD-1 agents for NSCLC treatment [69] inhibits their widespread use, especially in developing countries. Thus, although immunotherapy strategies have progressed, the chemotherapy agent docetaxel, with clearly demonstrated effects and good tolerability, will continue to have a positive effect on NSCLC treatment.

Chemotherapy agents for cancer treatment have benefits and harms. Most patients overestimate the intervention benefits and underestimate the harm associated with treatment [70]. Moreover, clinicians tend to underestimate the harm associated with medical treatments while overestimating their benefit [71]. Thus, assessments of adverse events associated with chemotherapy drugs are important for guiding the decision-making process in cancer treatment. Severe adverse events often result in treatment interruption or discontinuation and can even lead to hospitalization, disabilities and death. Information on adverse events can provide important reference material for clinical decision-making. Therefore, our systematic review and meta-analysis, which revealed that docetaxel is associated with an increased risk of severe infections, could provide clinicians with useful information to select the most appropriate treatment option for individual patients. Thus, our data should help guide treatment plans for NSCLC patients receiving docetaxel, with a positive effect on patient outcomes.

There are several strengths of our systematic review and meta-analysis. One, our study is the first systematic review and meta-analysis to assess the risk of severe infections (≥grade 3) in docetaxel-treated patients with NSCLC. Two, this systematic review and meta-analysis included 43 trials with 12,447 participants; these high numbers significantly increase the statistical power and ensure the reliability of the results. Three, we performed a subgroup analysis to differentiate studies testing docetaxel alone versus studies testing docetaxel-based combinations and found that docetaxel both alone and combined with other interventions is associated with an increased risk of severe infections.

Several limitations are inherent to our systematic review and meta-analysis. We did not perform a subgroup analysis according to tumor histology, performance status or race because of a lack of available data. In addition, the risk of severe infections associated with docetaxel was estimated in phase II and III randomized controlled trials; thus, the real risk to patients with comorbidities and a poor performance status may be higher. Moreover, most of the included trials were open-label studies, in which clinicians or other investigators could have easily known whether docetaxel led to severe infection, resulting in bias. Additionally, the heterogeneity was significant in our systematic review.

Our systematic review and meta-analysis suggest that docetaxel is significantly related to the risk of severe infections during the treatment of NSCLC patients. Early in the treatment process, clinicians should examine docetaxel-treated patients for any signs of infection in order to maximize the therapeutic benefit of this drug. Future research should explore the mechanism by which docetaxel leads to infection and determine ways to reduce this elevated risk of severe infections.

## METHODS

We reported this systematic review according to the Preferred Reporting Items for Systematic Reviews and Meta-Analyses (PRISMA) guidelines [72].

### Data sources and searches

We performed a literature search of the PubMed, EMBASE and Cochrane Central Register of Controlled Trials (CENTRAL) databases (up to February 28, 2017) without any language restrictions using the following terms: “carcinoma, non small cell lung”, “carcinomas, non-small-cell lung”, “lung carcinoma, non-small-cell”, “lung carcinomas, non-small-cell”, “non-small-cell lung carcinomas”, “nonsmall cell lung cancer”, “non-small- cell lung carcinoma”, “non small cell lung carcinoma”, “carcinoma, non-small cell lung”, “non-small cell lung cancer”, “non small cell lung cancer”, “docetaxel”, “docetaxel trihydrate”, “docetaxol”, “docetaxel anhydrous”, “N-debenzoyl-N-tert-butoxycarbonyl-10- deacetyltaxol”, “taxoltere metro”, “taxotere”, “NSC 628503”, “RP 56976”, “RP-56976”, “random*” and “randomized controlled trial”. Human subject and clinical trial restrictions were implemented in the searches. We also searched the www.clinicaltrials.gov website to access unpublished data. Moreover, we checked each reference listed in the included studies, relevant reviews, and guidelines to include any previously ignored papers. All titles and abstracts from the initial search were transferred to Endnote X7 software.

### Study selection

The study selection was performed using Endnote X7 software. We selected eligible trials according to the following criteria: (1) participants were patients with NSCLC; (2) participants were grouped into a docetaxel- treated group (with or without other interventions) and a control group (without docetaxel); (3) severe infection (≥grade 3) was a measured outcome; and (4) study design was a randomized controlled trial. The exclusion criteria consisted of the following: (1) duplicate studies and studies without outcomes or original data; and (2) trials with no events in both the docetaxel group and control group. For publications of the same trial or patient cohort, we chose the latest publication with the most complete data.

### Risk of bias assessment

The risk of bias of each included trial was independently assessed by two authors using the risk of bias assessment tool in Review Manager 5.3 software according to the Cochrane Handbook for Systematic Reviews of Interventions version 5.1 [73]. Six aspects were evaluated, including selection bias (random sequence generation and allocation concealment), performance bias (blinding of participants and personnel), detection bias (blinding of outcome assessment), attrition bias (incomplete outcome data), reporting bias (selective reporting) and other bias. Each aspect included three options (low risk of bias, high risk of bias, and unclear risk of bias) from which the authors could choose according to the content of the trial. All inconsistencies in this process were resolved by discussion.

### Data extraction

In this systematic review and meta-analysis, infection was defined as a disease characterized by febrile neutropenia, non-specified infection, pneumonia or sepsis as reported by the trial. Severe infections (≥grade 3) were graded using the National Cancer Institute Common Toxicity Criteria version 2 or version 3. We created a standardized form using Microsoft Excel 2013 to extract data from all included studies. The data were independently extracted by two authors using the standardized form. We entered the following data into the form: the first author’s name, publication year, sample size, description of patients (age, sex, and race), type of intervention, dose, trial phase, disease stage, tumor histology, severe infection (≥grade 3) events and median follow-up. Additionally, we contacted the authors of the article when we encountered any unclear information. We resolved all disagreements in this process through discussion.

### Data analysis

We performed all statistical analysis using Stata 14.0 software. Severe infection events were considered dichotomous data and were pooled using the RR and 95% CI. We judged significant results according to the *P*-value; a *P*-value less than 0.05 indicated a significant result. We assessed heterogeneity among included studies using the *I^2^* statistic. If significant heterogeneity was present (*I^2^*>50%), we performed a meta-analysis using the random effects model [73]. Otherwise, we selected the fixed effects model. Furthermore, subgroup analyses were conducted according to age, type of intervention, docetaxel dosage, and trial phase. Sensitivity analysis was performed to determine whether an individual study influenced the overall result. We assessed the influence of each study on the pooled results by sequentially deleting a single study. Meta- regression was performed to investigate any potential covariates with a substantial impact on the heterogeneity among studies. We performed a meta-regression according to the following potential covariates: age, type of intervention, docetaxel dosage, and trial phase. In addition, we used Begg’s test to examine publication bias, which is recommended by the Cochrane Handbook for Systematic Reviews of Interventions [73].

## SUPPLEMENTARY MATERIALS



## Abbreviations

RR: relative risk
CI: confidence interval
NSCLC: non-small cell lung cancer.
