# Supplementary material for: Docetaxel increases the risk of severe infections in the treatment of non-small cell lung cancer: a meta-analysis
Source: Oncoscience. 2018 Aug 22;5(7-8):220–38. doi: 10.18632/oncoscience.444 (PMC6142895; doi:10.18632/oncoscience.444)
Supplement: Supplementary file 1 [file oncoscience-05-220-s001.pdf]

SUPPLEMENTARY MATERIALS

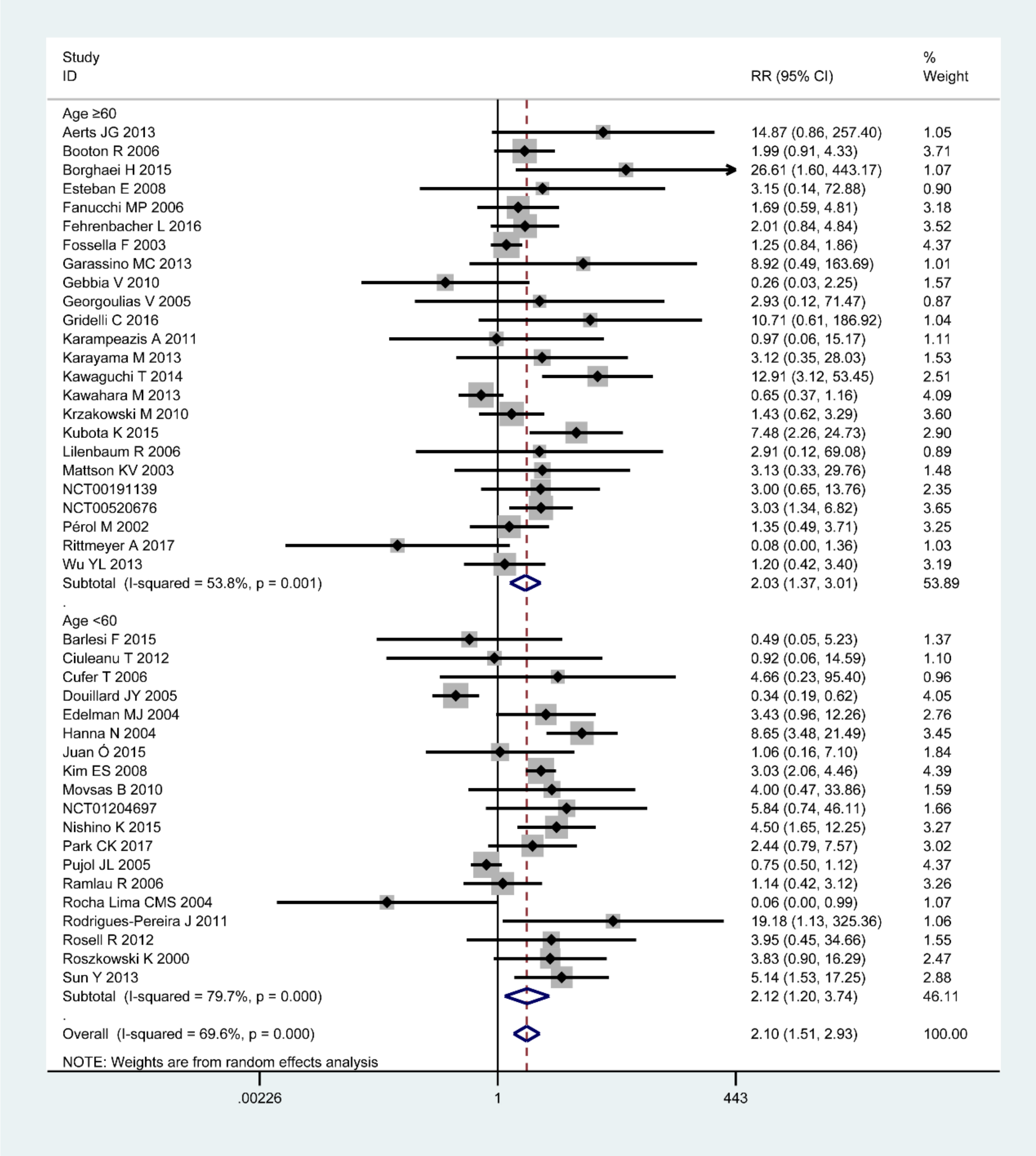

Supplemental Figure 1: Subgroup analysis by age.

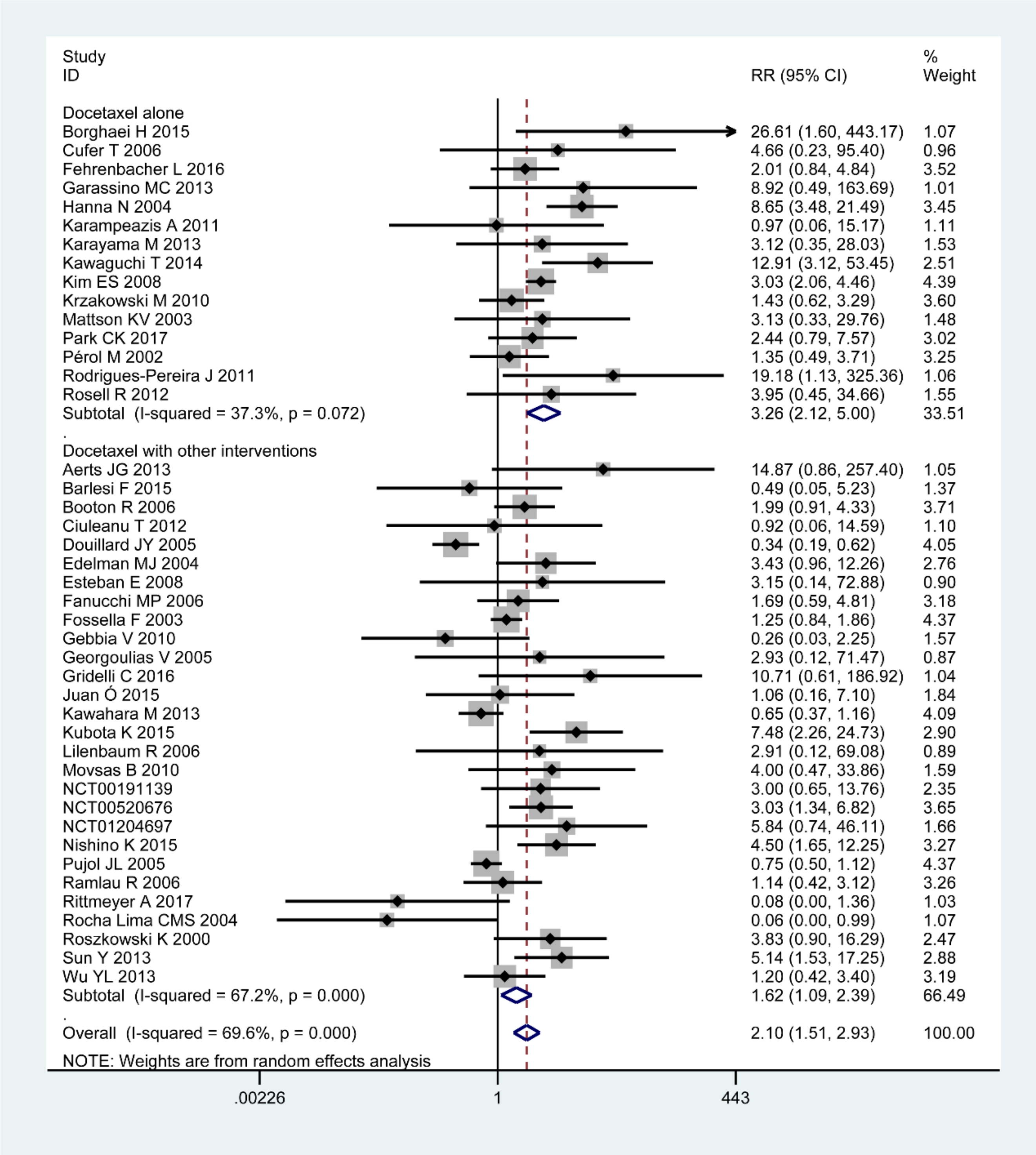

Supplemental Figure 2: Subgroup analysis by the type of intervention.

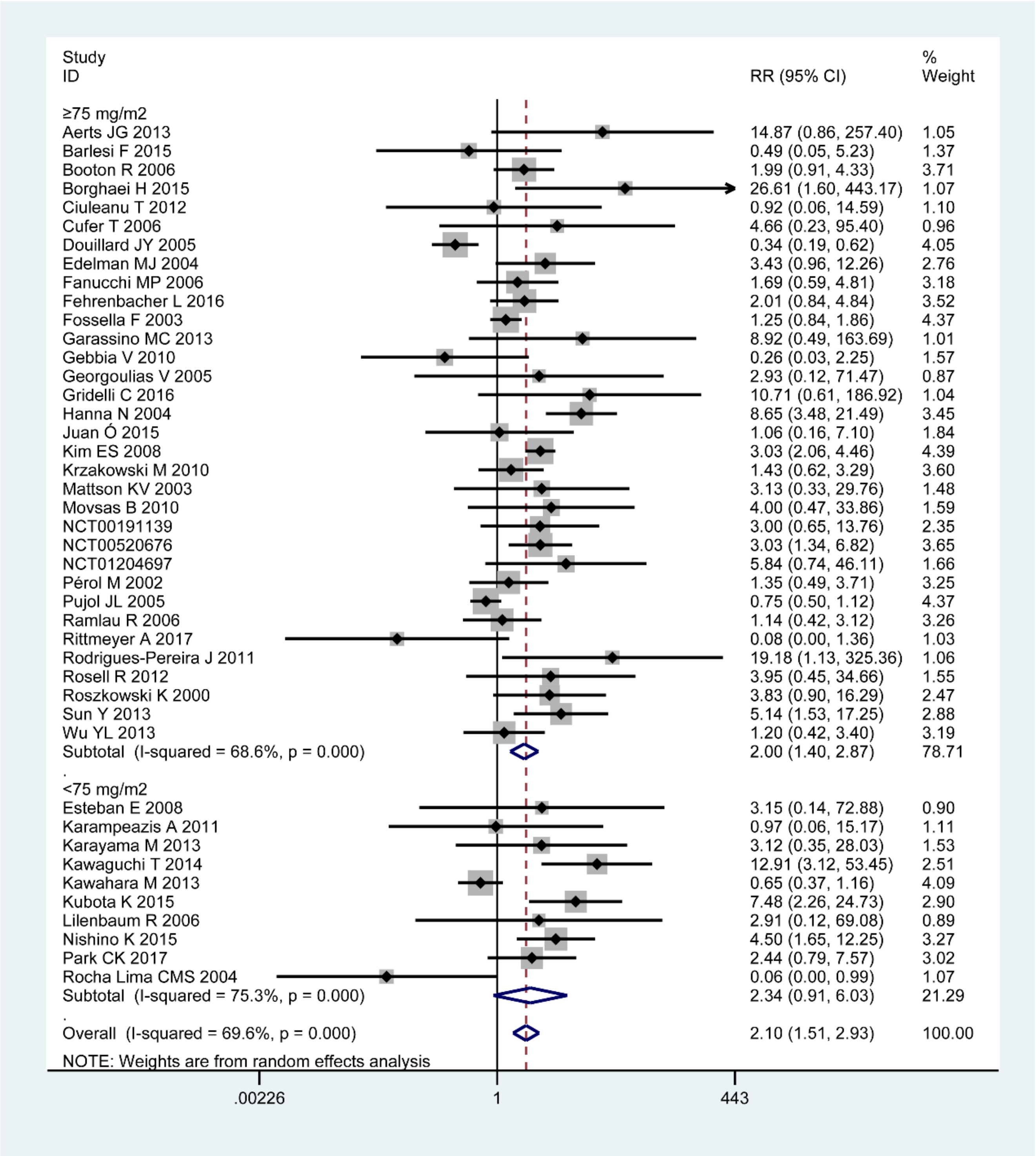

Supplemental Figure 3: Subgroup analysis by docetaxel dosage.

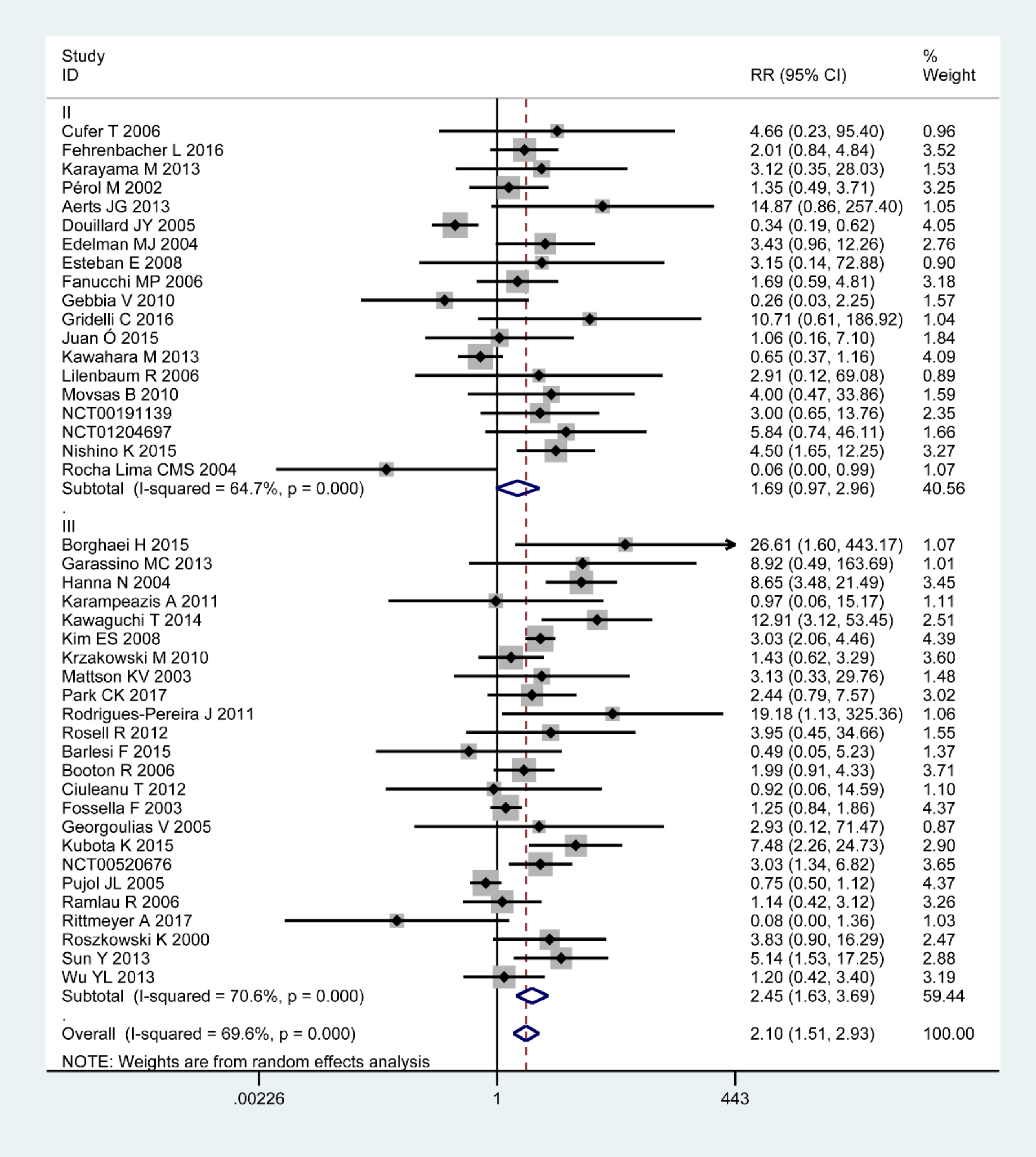

Supplemental Figure 4: Subgroup analysis by trial phase.

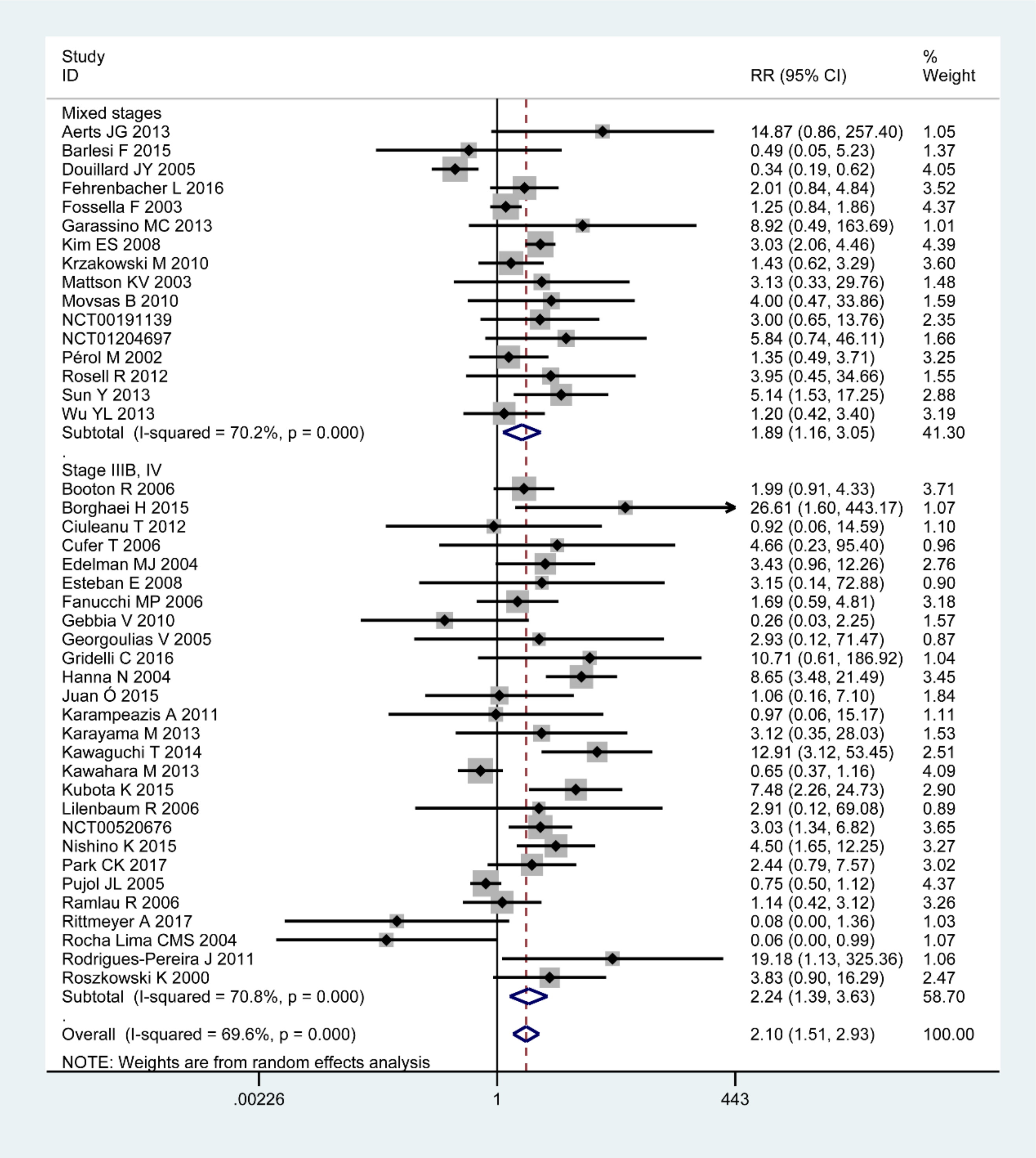

Supplemental Figure 5: Subgroup analysis by disease stage.
